# 3-Decylcatechol induces autophagy-mediated cell death through the IRE1α/JNK/p62 in hepatocellular carcinoma cells

**DOI:** 10.18632/oncotarget.17732

**Published:** 2017-05-09

**Authors:** Da-Hye Go, Yu Geon Lee, Da-Hye Lee, Jin-A Kim, In-Hwa Jo, Yeon Soo Han, Yong Hun Jo, Kwang-Youn Kim, Young-Kyo Seo, Jae-Hak Moon, Chang Hwa Jung, Tae-Il Jeon

**Affiliations:** ^1^ Department of Animal Science, College of Agriculture & Life Science, Chonnam National University, Gwangju, Republic of Korea; ^2^ Research Group of Metabolic Mechanism, Korea Food Research Institute, Seongnam, Republic of Korea; ^3^ Department of Food Biotechnology, Korea University of Science and Technology, Seongnam, Republic of Korea; ^4^ Division of Plant Biotechnology, Institute of Environmentally-Friendly Agriculture (IEFA), College of Agriculture and Life Sciences, Chonnam National University, Gwangju, Republic of Korea; ^5^ School of Life Sciences, Ulsan National Institute of Science and Technology, Ulsan, Republic of Korea; ^6^ Department of Food Science & Technology, Chonnam National University, Gwangju, Republic of Korea

**Keywords:** autophagy, cell death, ER stress, hepatocellular carcinoma, urushiol

## Abstract

The natural, phenolic lipid urushiol exhibits both antioxidant and anticancer activities; however, its biological activity on hepatocellular carcinoma (HCC) has not been previously investigated. Here, we demonstrate that an urushiol derivative, 3-decylcatechol (DC), induces human HCC Huh7 cell death by induction of autophagy. DC initiates the autophagic process by activation of the mammalian target of rapamycin signaling pathway via Unc-51-like autophagy activating kinase 1, leading to autophagosome formation. The autophagy inhibitor, chloroquine, suppressed autolysosome formation and cell death induction by DC, indicating an autophagic cell death. Interestingly, DC also activated the endoplasmic reticulum (ER) stress response that promotes autophagy via p62 transcriptional activation involving the inositol-requiring enzyme 1α/c-Jun N-terminal kinase/c-jun pathway. We also show that cytosolic calcium mobilization is necessary for the ER stress response and autophagy induction by DC. These findings reveal a novel mechanism by which this urushiol derivative induces autophagic cell death in HCC.

## INTRODUCTION

Hepatocellular carcinoma (HCC) is one of the most prevalent tumor types worldwide. HCC is a major cause of cancer-related mortality because of its aggressive malignancy and late diagnosis. Therapeutic strategies for HCC include surgical resection, ablation, liver transplantation, and systemic or infusional chemotherapy. However, the five-year survival rate for patients with liver cancer is still less than 14%, overall [1]. Although chemotherapy using sorafenib is a critical therapeutic approach for patients with HCC, the drug only improves survival in advanced HCC [2]; therefore, identification of new, effective chemotherapeutic agents is needed, particularly those that allow earlier treatment.

Urushiol is a major component of the sap of the lacquer tree (*Toxicodendron vernicifluum*, Anacardiaceae) that has been used as a traditional, natural curative resin in East Asia. Urushiol is a mixture of structurally similar catechol derivatives that have a saturated, or unsaturated, long alkyl side chain [3]. Recent studies have shown that urushiol exhibits anticancer activity against various types of cancer cells [4–6]; however, the underlying molecular mechanism is poorly understood.

Autophagy is a catabolic process whereby macromolecules and organelles are sequestered in double membrane vesicles (autophagosomes) and are degraded and recycled following lysosomal fusion [7]. In response to a variety of cellular states, the inhibition of the mammalian target of rapamycin complex 1 (mTORC1) initiates autophagosome formation in a process requiring the sequential action of several autophagy-related proteins (Atg) involved in phagophore nucleation, elongation, and degradation mechanisms, thereby maintaining nutrient and energy homeostasis. This process occurs constitutively, but is also induced in response to cellular stresses such as starvation, damage to proteins and organelles, or infection by pathogen [8]. Given its fundamental role in cell survival, it is not surprising that autophagy protects cancer cells from metabolic stress and anticancer therapy. However, increasing evidence also supports the role of autophagy in tumor suppression [9]. For example, loss of an essential autophagy gene such as beclin-1 has been found in various human cancers including HCC [10]. Several studies have shown that mTOR inhibitors that are able to induce autophagy inhibit liver tumor growth *in vitro* and in a mouse model [11]. Hence, small molecule autophagy inducers would seem to offer potential as treatments for HCC.

Autophagy is strongly induced by the unfolded protein response (UPR) that is triggered by perturbation of endoplasmic reticulum (ER) functions including protein folding, Ca^2+^ storage, and lipid synthesis [12]. Recent studies have shown that the protein kinase RNA-like endoplasmic reticulum kinase (PERK)/eIF2α pathway increases transcription of autophagy related genes during ER stress [13]. In addition, activation of inositol-requiring enzyme 1α (IRE1α)/c-Jun N-terminal kinase (JNK) pathway is also able to induce activation of beclin-1 and autophagy [14]. However, the link between autophagy and UPR is complex and remains unclear.

In this study, we explored the mechanisms leading to cell death in HCC induced by urushiol and its derivatives. We found that 3-decylcatechol (DC) induced autophagic flux by increasing p62/SQSTM1 expression through the IRE1α/JNK/c-jun pathway and by suppression of mTOR signaling, promoting autophagic cell death. Moreover, we showed that an increase in intracellular calcium levels is associated with DC-induced ER stress and autophagy. These findings provide evidence of the potential of DC as a therapeutic agent for HCC.

## RESULTS

### DC-induced cell death is associated with autophagic processes in Huh7 cells

**Figure 1:**
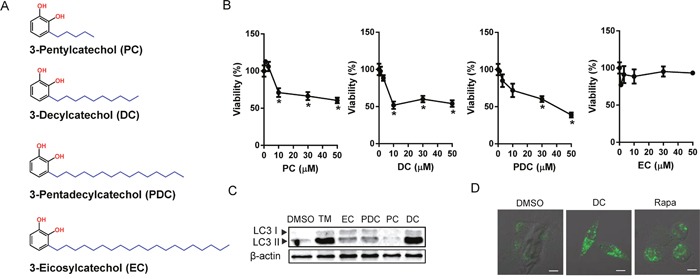
Effects of urushiol derivatives on cell death and autophagosomal marker in human hepatocellular carcinoma cells **(A)** The structure of urushiol derivatives. **(B)** Huh7 cells were treated with various concentrations of urushiol derivatives for 48h. The cell viability was measured by MTT assay. *P <0.05 vs. control DMSO **(C)** Immunoblotting for LC3 from lysates of cells treated with 10 μM urushiol derivatives or 10 μg/ml tunicamycin (TM). **(D)** Cells in 8-well chamber slides were treated with 10 μM DC or 50 nM rapamycin (Rapa) for 24h. A representative immunofluorescence images with LC3 antibody staining. Scale bar = 10 μm.

Urushiol derivatives (PC, DC, PDC, and EC) were synthesized as reported in a previous study [3] (Figure 1A). Initially, we compared the cytotoxic effects of each of the urushiol derivatives on the human HCC cell line Huh7 by MTT assay. Cells were treated with a range of concentrations from 0 to 50 μM of PC, DC, PDC, or EC for 48 h. As shown in Figure 1B, DC had the most potent effect on Huh7 HCC cell viability. Consistently, among the urushiol derivatives, DC markedly increased the conversion of LC3-I to LC3-II, an indicator of the autophagic process (Figure 1C). Upon induction of autophagy, changes in the localization of LC3 into the autophagosomal membrane could be detected as punctate, immunostained foci [15]. As indicated in Figure 1D, endogenous LC3 staining was detected as multiple punctate structures in rapamycin or DC-treated cells but not in DMSO-treated control cells, suggesting that autophagy is associated with DC-induced Huh7 cell death.

### DC-induced autophagy promotes necrotic cell death of Huh7 cells

**Figure 2:**
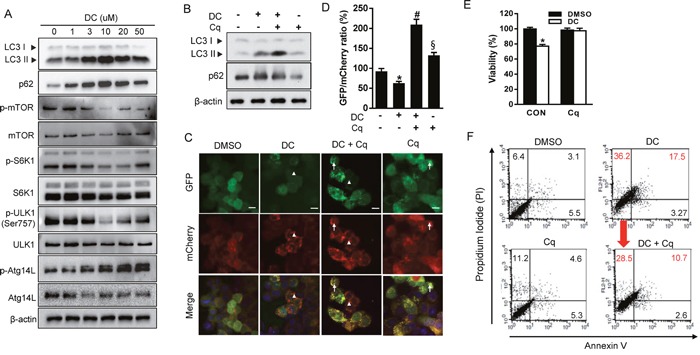
DC represses mTOR signaling and inhibition of autophagy prevents DC-induced cell death **(A)** Cells were treated with increasing concentration of DC for 24h. Cell lysates were analyzed for LC3, p62, mTOR, p-mTOR (Ser2448), S6K1, p-S6K1 (Thr389), ULK1, p-ULK1 (Ser757), ATG14L1, or p-ATG14L (Ser29) by immunoblotting. **(B)** Cells were treated with 10 μM DC and/or 10 μM chloroquine (Cq). Cells lysates were analyzed for LC3 or p62 by immunoblotting. **(C)** mCherry-GFP-LC3 stable Huh7 cells in 8-well chamber slides were treated with 10 μM DC and/or 10 μM Cq for 24h. A representative microscopy image showing red-colored autolysosomes (arrowheads) and yellow-colored autophagosomes (arrows). Scale bar = 10 μm. **(D)** Quantification of mCherry-GFP-LC3 puncta. The data are presented as percentage of GFP/mCherry ratio. Data are mean ± SEM; n=10. *P <0.05 vs. control DMSO; ^#^*P* <0.05 vs. DC; ^§^*P* <0.05 vs. DC + Cq. **(E)** MTT assay and **(F)** Annexin V-FITC/PI double staining in cells treated with 10 μM DC and/or 10 μM Cq for 24h. Data are mean ± SEM; n=3. *P <0.05 vs. control DMSO.

The unc-51-like autophagy activating kinase 1 (ULK1), a mammalian counterpart of yeast ATG1, controls a key step in the early triggering of autophagy, and it is negatively regulated by mTOR signaling [16]. ULK1 activation is especially crucial for the initiation of phagophore nucleation mediated by ATG14L-containing VPS34 lipid kinase complexes [17]. We therefore examined whether DC stimulated the mTOR-ULK1 pathway to induce autophagy (Figure 2A). DC treatment of HCC cells resulted in a notable inhibition of the phosphorylation of mTOR and p70S6 kinase (S6K1, a known mTORC1 substrate) in a dose-dependent manner. In addition, DC-mediated inhibition of mTOR leads to dephosphorylation (Ser757) and activation of ULK1, as demonstrated by monitoring the phosphorylation of ATG14L at Ser29, suggesting that DC initiates autophagy via the mTOR/ULK1 pathway. Moreover, knockdown of ULK1 suppressed DC-induced cell death (Supplementary Figure 1).

We next determined whether DC treatment induces autophagosome formation or blocks their clearance. LC3 conversion was monitored in the presence of chloroquine (Cq), which blocks lysosome acidification, degradation of autophagosome contents, and autophagic flux. DC significantly increased the level of LC3-II, which was elevated to a greater extent in the presence of Cq (Figure 2B). Alternatively, autophagic flux can be assessed using the mCherry-GFP-LC3 reporter protein, which displays yellow fluorescence (green merged with red) in nonacidic autophagosomes, and red fluorescence in autolysosomes due to the quenching of GFP in the acidic lysosome [15]. Huh7 cells stably expressing mCherry-GFP-LC3 were treated with DC, Cq, or both. The number of mCherry-only puncta were significantly increased in DC-treated cells compared to that in control cells, and this effect was suppressed by the addition of lysosomal inhibitor Cq (Figure 2C and 2D), indicating that DC induces fusion of the autophagosome and lysosome to form an autolysosome. In addition, inhibition of autophagic flux with Cq completely blocked the DC-induced reduction of HCC cell viability (Figure 2E). Moreover, flow cytometry analysis revealed that DC treatment resulted in a significant increase of propidium iodide-positive but annexin V-negative cells in the population, a sign of necrotic cell death, which decreased in cells treated with Cq (Figure 2F). Necrotic markers, lactase dehydrogenase (LDH) and heat shock protein 90 (HSP90), were also remarkably increased by DC, which were completely reversed with Cq (Supplementary Figure 2). Altogether, these results demonstrate that DC increases autophagic initiation and flux, thereby leading to necrotic cell death in HCC cells.

### DC-induced cell death is mediated by p62-dependent autophagy in Huh7 cells

**Figure 3:**
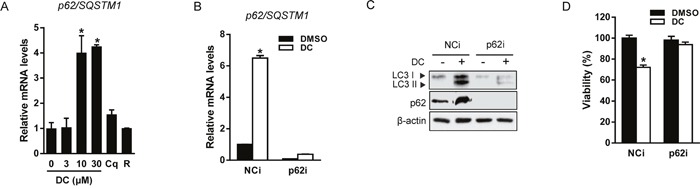
DC induces p62-dependent autophagy **(A)** Cells were treated with 10 μM DC, 10 μM Cq, or 50 nM rapamycin (R) for 24h. mRNA level for p62/SQSTM1 was analyzed by RT-qPCR. *P <0.05 vs. control DMSO. (**B** and **C**) Cells were transfected with siRNA (10 nM) for p62 or negative control (NCi) for 24h, and then 10 μM DC was added and cells were harvested after 24h. mRNA level for p62/SQSTM1 was analyzed by RT-qPCR and cell lysates were analyzed for LC3 or p62 by immunoblotting. **(D)** Cell viability was measured by MTT assay. *P <0.05 vs. NCi DMSO. Data are mean ± SEM; n=3.

The autophagic adaptor p62/SQSTM1 (sequestosome1) targets proteins of the autophagosome cargo which bind, linking them to the autophagic machinery and enabling the degradation of selective substrates such as misfolded proteins, damaged organelles, and microbes [18]. Because p62 is incorporated into autophagosomes and degraded upon induction of autophagy, it is widely used as a marker of autophagic flux. However, we found that both mRNA and protein expression of p62 were significantly increased by DC in a dose-dependent manner, although autophagy flux increased (Figure 2 and 3A). In addition, the p62 mRNA level was not changed by treatment with Cq (autophagy inhibitor) or rapamycin (autophagy inducer), suggesting that DC could induce stress-mediated autophagy. In order to investigate whether p62 is involved in DC-induced autophagy and cell death, we knocked down the expression of the p62 gene using siRNA in Huh7 cells (Figure 3B). As shown in Figure 3C and 3D, p62 silencing significantly suppressed LC3-II and p62 accumulation, and cell death in response to DC, thereby indicating that the upregulation of p62 transcription is required for DC-induced autophagic cell death.

### DC-elicited ER stress induces autophagy in Huh7 cells

**Figure 4:**
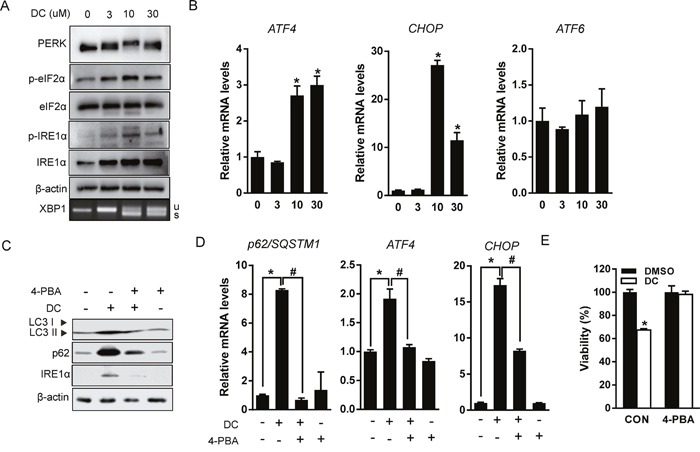
DC induces ER stress-mediated autophagy **(A)** Cells were treated with increasing concentration of DC for 24h. Cell lysates were analyzed for PERK, eIF2α, p-eIF2α (Ser51), IRE1α, or p-IER1α (Ser724) by immunoblotting, and XBP1 splicing was analyzed by RT-PCR. **(B)** mRNA levels for ATF4, CHOP, or ATF6 were analyzed by RT-qPCR. *P <0.05 vs. control DMSO. **(C)** Cells were pretreated with 2mM 4-phenylbutyric acid (4-PBA) for 1h and then exposed to 10 μM DC for 24h. LC3, p62, or IRE1α protein levels were analyzed by immunoblotting. **(D)** p62, ATF4, or CHOP mRNA levels were analyzed RT-qPCR. *P <0.05 vs. control DMSO; ^#^*P* <0.05 vs. DC. **(E)** Cell viability was analyzed by MTT assay. *P <0.05 vs. control DMSO. Data are mean ± SEM; n=3.

Recent studies have shown that p62 expression is upregulated by stress conditions including starvation [19], oxidative stress [20], and the accumulation of dysfunctional proteins [13]. UPR signaling is mediated by three ER membrane-bound proteins, PERK, IRE1α, and activating transcription factor 6 (ATF6). These three initiators, all activate transcriptional programs mediated by distinct transcription factors such as ATF4, spliced X-box binding protein 1 (XBP1), and cleaved ATF6 [21]. Therefore, we hypothesized that UPR signaling could participate in the transcriptional activation of p62 by DC treatment. As shown in Figure 4A and 4B, DC elevated phosphorylation of PERK and its downstream target, eIF2α, resulting in upregulation of mRNA expression of ATF4 and CCAAT-enhancer-binding protein homologous protein (CHOP). DC treatment also significantly increased phosphorylation of IRE1α and the splicing of XBP-1, but not ATF6 expression. Moreover, the chemical chaperone and ER stress inhibitor 4-phenylbutyric acid (4-PBA) blocked DC-induced UPR activation, including ATF4 and CHOP upregulation, DC-induced LC3 conversion, and upregulation of p62 expression, which were all markedly reversed (Figure 4C and 4D). 4-PBA treatment also significantly restored the cytotoxicity of DC in Huh7 cells (Figure 4E). These results suggest that DC-elicited ER stress can induce p62-dependent autophagy, and that UPR activation is essential for autophagic cell death in Huh7 cells.

### DC upregulates p62 expression through the IRE1α/JNK pathway in Huh7 cells

**Figure 5:**
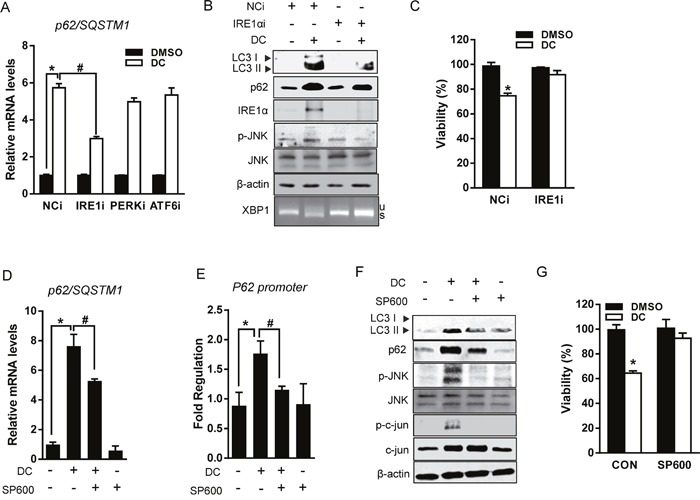
DC upregulates p62 transcription via IRE1/JNK/c-jun axis **(A)** Cells were transfected with siRNA (10 nM) for PERK, IRE1α, ATF6, or negative control (NCi) for 24h, and then 10 μM DC was added and cells were harvested after 24h. mRNA level for p62 was analyzed by RT-qPCR. *P <0.05 vs. NCi DMSO; ^#^*P* <0.05 vs. NCi DC. **(B)** Cell lysates were analyzed for LC3, p62, JNK, p-JNK (Thr183/Tyr185), or IRE1α by immunoblotting. **(C)** Cell viability was measured by MTT assay. *P <0.05 vs. control DMSO. **(D)** Cells were pretreated with 20 μM SP600125 (SP600) for 1h and then exposed to DC for 24h. mRNA level for p62 was analyzed by RT-qPCR. *P <0.05 vs. control DMSO; ^#^*P* <0.05 vs. DC. **(E)** Reporter assays were performed using human p62 promoter construct (-1,650 to +120). Huh7 cells were transfected with pGL3-p62 luciferase reporter for 24h before treatment with 10 μM DC and/or 20 μM SP600 for 24h. *P <0.05 vs. control DMSO; ^#^*P* <0.05 vs. DC. **(F)** LC3, p62, JNK, p-JNK (Thr183/Tyr185), Jun, or p-c-Jun (Ser63) protein levels were analyzed by immunoblotting. **(G)** Cell viability was analyzed by MTT assay. *P <0.05 vs. control DMSO. Data are mean ± SEM; n=3.

Next, we examined which branch of the UPR signaling pathway is important for the transcriptional induction of p62 in HCC cells. Cells were knocked down with siRNA against each UPR branch and the mRNA expression of p62 was measured. As expected, transfection with siRNA successfully reduced the mRNA expression of each of the branches and suppressed DC activation of UPR, including mRNA expression of IRE1α and PERK (Supplementary Figure 3). Interestingly, DC-induced p62 expression was significantly reduced in IRE1α-silenced cells but not in PERK- and ATF6-silenced cells (Figure 5A). Similar to the effect produced by 4-PBA, IRE1α silencing also restored accumulation of p62 and LC3-II and Huh7 cell death induced by DC addition (Figure 5B and 5C), indicating that DC-induced p62-dependent autophagy is mediated by activation of IRE1α signaling. We subsequently investigated whether downstream transcription factors of IRE1α increase transcriptional activation of p62. Although spliced XBP1 was markedly increased by DC, and moderated by IRE1α siRNA (Figure 5B), mRNA expression of p62 was not reduced in XBP1-silenced cells (Supplementary Figure 4). Since it is known that ATF4 and nuclear factor, erythroid 2-like 2 factor (NRF2) increase transcription of p62 in response to stress [13, 20], we also verified, by means of siRNA, that ATF4 and NRF2 was not involved in DC induction of p62 expression (Supplementary Figure 4).

In addition to catalyzing XBP1 splicing, IRE1α activates the stress-induced JNK pathway, which contributes to the death of ER-stressed cells [22]. Indeed, DC phosphorylation of JNK was suppressed in IRE1α knockdown cells (Figure 5B). We, therefore, examined if DC-induced p62 transcriptional activation could be inhibited by the JNK inhibitor SP600125 (SP600). As shown in Figure 5D and 5E, SP600 reduced the mRNA expression of p62 through the inhibition of promoter activity, which was increased in DC-treated Huh7 cells. As expected, DC treatment significantly induced the c-jun phosphorylation, which is blocked by SP600 (Figure 5F), suggesting that transcription factor c-jun might be involved in DC transcriptional activation of p62. Furthermore, DC-induced cell death was also abolished in SP600-treated Huh7 cells (Figure 5G) as well as in JNK1- or JNK2-deficient MEF (Supplementary Figure 5). Taken together, these results demonstrate that DC-related autophagic cell death induced via the upregulation of p62 transcription is dependent on the IRE1α/JNK pathway in Huh7 cells.

### DC increases intracellular calcium concentrations leading to ER stress and autophagy in Huh7 cells

**Figure 6:**
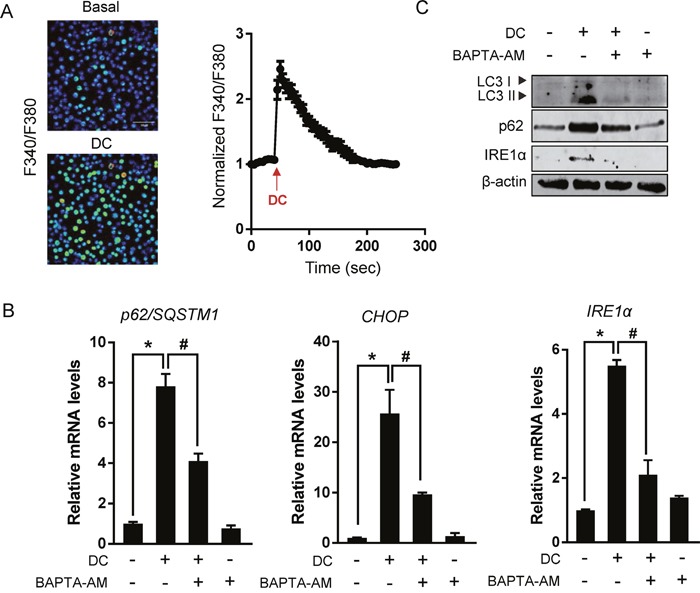
Cytosolic Ca^2+^ elevation is required for DC action **(A)** Left panel: Images of cells loaded with Fura-2/AM show fluorescence gray levels in pseudocolor localized photo excitation. Right panel: Representative traces show Ca^2+^ responses measured by changes in the fluorescence ratio (F340/F380 nm). Arrows indicate the time of 10 μM DC application. Data are mean ± SEM; n=30. **(B)** Cells were pretreated with 10 μM BAPTA-AM for 1h and then exposed to 10 μM DC for 24h. mRNA level for p62, CHOP, or IRE1α was analyzed by RT-qPCR. *P <0.05 vs. control DMSO; ^#^*P* <0.05 vs. DC. **(C)** LC3, p62, or IRE1α protein levels were analyzed by immunoblotting. Data are mean ± SEM; n=3.

Aberrant calcium regulation in the ER is known to cause protein unfolding due to the dysfunction of Ca^2+^-dependent molecular chaperones, thereby triggering the UPR and autophagy [12]. Therefore, in order to test whether the addition of DC can increase intracellular Ca^2+^ levels, ratiometric Ca^2+^ imaging using Fura-2/AM was conducted and monitored using fluorescence microscopy. The treatment of Huh7 cells with DC generated a strong release of calcium and consequent transient Ca^2+^ spike (Figure 6A). To further test whether the mechanism of ER stress and autophagy induced by DC was calcium-dependent, we employed the cytosolic calcium chelator BAPTA-AM. As shown in Figure 6B and 6C, BAPTA-AM significantly reduced increased CHOP and IRE1α expression and accumulation of p62 and LC3-II in DC-treated Huh7 cells, suggesting that calcium dysregulation causes the ER stress and autophagy induced by DC. Interestingly, this phenomenon was not observed in EGTA-treated cells, indicating that Ca^2+^ influx from outside the cell was not involved (Supplementary Figure 6).

## DISCUSSION

Autophagy is often characterized as having a pro-survival role in cancer cells, but accumulating evidence also demonstrates an active contribution of autophagy to cell death [23]. In this study, we demonstrated that urushiol derivative DC increased both autophagosome formation and flux in HCC cells, and that inhibition of autophagy by chloroquine completely abolished DC-induced necrotic cell death, thereby suggesting that DC-induced autophagy promotes HCC cell death. Our data also demonstrate that DC inhibits the mTOR signaling pathway, thereby activating autophagy-initiating kinase ULK1 and a component of nucleation complex ATG14L. As mTOR signaling negatively regulates autophagy induction and is hyperactivated in 40-50% of HCC cases [24], prevention of HCC by mTOR inhibition may involve autophagy. Several studies have shown that genetic and pharmacological inhibition of the mTOR signaling pathway causes HCC cell death associated with autophagy [11]. Thus, our results suggest DC-induced autophagy and cell death are positively connected in HCC cells.

The autophagy-associated protein, p62, acts as a cargo receptor for degradation of ubiquitinated substrates. It is localized to autophagosomes by LC3 binding and is itself degraded by autophagy [25]. However, we unexpectedly found a remarkable upregulation of p62 mRNA and protein level in DC-treated HCC cells. Moreover, p62 silencing reversed LC3 induction and cell death by DC. Consistent with our findings, two recent studies demonstrated that resveratrol and dehydroepiandrosterone induce autophagic cell death through the upregulation of p62 gene expression in chronic myelogenous leukemia cells [26] and human hepatoma cell line HepG2 [27], respectively.

Increasing evidence supports a mechanistic link between UPR and autophagy in cancer therapy [28]. In addition to the role of p62-dependent autophagy, our data also show that ER stress-elicited UPR activation is responsible for the effect of DC on autophagy and cell death. Interestingly, DC-induced upregulation of p62 gene expression and LC3-II conversion were reduced by silencing of one of the UPR branches, IRE1α, but not by silencing of PERK or ATF6. The promoter of the p62 gene is activated by various transcription factors such as NRF2 [20], NFκB (nuclear factor of kappa light polypeptide gene enhancer in B-cells) [29], PDEF (Prostate-derived Ets factor) [30], and AP-1 (activator protein 1) [26]. Recent studies reported that UPR-associated transcription factors ATF4 [13] and XBP1 [31] are also implicated in ER stress-induced p62 gene expression. However, the increase of p62 mRNA expression by DC was not repressed by knockdown of ATF4 and XBP1 in our model. While IRE1α RNAse is implicated in XBP1 splicing and mRNA decay, its kinase domain is linked with other stress-induced pathways such as JNK and NFκB [22]. Studies performed with JNK inhibitor and JNK1/2 knockout cells showed that the JNK signaling pathway is required for the upregulation of p62 transcription via its promoter activation and for the induction of autophagy and cell death by DC. These results suggest a connection between ER stress and autophagy through the IRE1α/JNK/p62 in DC-elicited HCC cell death.

Finally, DC increases intracellular Ca^2+^ concentrations and cytosolic calcium chelator BAPTA-AM suppresses UPR activation and autophagy remarkably, thereby suggesting that cytosolic Ca^2+^ mobilization is required for the ER stress response and p62-dependent autophagy by DC. Since cytosolic Ca^2+^ also activates autophagy via the calcium-dependent AMPK/mTOR pathway [32], further studies are required to understand the detailed regulatory mechanism governing the effect of DC on the initiation of autophagy, including mTOR inhibition, and ULK1 and ATG14L activation.

**Figure 7:**
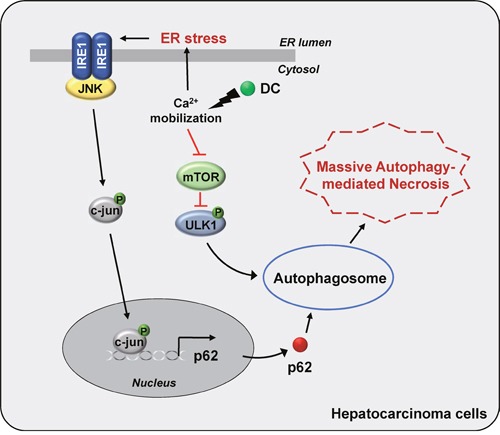
A model for the proposed mechanism of DC-induced cell death DC triggers a rise in cytosolic Ca^2+^ mobilization, which stimulates ER stress-evoked IRE1/JNK/c-jun pathway, leading to autophagy via upregulation of p62 transcription. DC-elicited mTOR inhibition, which activates ULK1 pathway, initiating autophagosome formation. Thus, DC-induced massive autophagic flux promotes HCC cell death.

In conclusion, we have shown that the stimulation of HCC cells with the urushiol derivative DC induces p62 transcription through the IRE1α/JNK/c-jun pathway involving a rise in cytosolic Ca^2+^ and autophagosome formation through the mTOR/ULK1 signaling pathway, thereby promoting cell death (Figure 7). Our study suggests a possible application of urushiol and its derivatives as chemotherapeutic agents for the treatment of HCC.

## MATERIALS AND METHODS

### Chemicals and antibodies

Four urushiol derivatives 3-pentylcatechol (PC), 3-decylcatechol (DC), 3-pentadecylcatechol (PDC), and 3-eicosylcatechol (EC) were synthesized as reported in a previous study [3] (Figure 1A). 3-(4,5-dimethylthiazol-2-yl)-2,5-diphenyltetrazolium bromide (MTT), tunicamycin, rapamycin, chloroquine (Cq), and 4-phenylbutyric acid (4-PBA) were purchased from Sigma-Aldrich. SP600125 (SP600) and BAPTA-AM were purchased from Calbiochem. Antibodies against mTOR, p-mTOR (Ser2448), p-S6K1 (Thr389), S6K1, p-ULK1 (Ser757), ULK1, PERK, eIF2α, p-eIF2α (Ser51), IRE1α, c-Jun, p-c-Jun (Ser63), JNK, p-JNK (Thr183/Tyr185) and LC3A/B were purchased from Cell Signaling Technology. Polyclonal rabbit anti-LC3B, p-IER1α (Ser724), and anti-p62 were purchased from Novus Biologicals and Sigma-Aldrich, respectively. Anti-β-actin, anti-HSP90, and HRP-conjugated secondary antibodies were purchased from Santa Cruz Biotechnology. Polyclonal rabbit anti-p-ATG14L (Ser29) and anti-ATG14L was generated as previously described [17].

### Cell culture and RNA interference

Human hepatocellular carcinoma Huh7 cell line was purchased from the American Type Culture Collection (ATCC). Retroviral pBABE-puro mCherry-EGFP-LC3B was purchased from Addgene (Plasmid #22418, deposited by Jayanta Debnath). Briefly, the retroviral vector was transfected into 293T cells (ATCC) with pCMV-VSV-G (Addgene, #8454) and pUMVC (Addgene, #8449) using FuGENE6^®^ reagent (Roche Applied Science). Collected viruses were infected into Huh7 cells. JNK1- and 2-knockout MEF cells were kindly provided by Dr. Zigang Dong (University of Minnesota, Austin, MN, USA). The cells were maintained in Dulbecco's modified Eagle's medium (HyClone^TM^, GE Healthcare) supplemented with 10% heat-inactivated fetal bovine serum (Atlas Biologicals) and antibiotics (Invitrogen) in an atmosphere of 5% CO_2_ at 37°C. The siRNA targeting human p62/SQSTM1, IRE1α, PERK, ATF6, ATF4, NRF2, and XBP1 and siRNA negative control were purchased from Santa Cruz Biotechnology. Huh7 cells were transfected for 24h with 10 nM of each siRNA by Lipofectamine^®^ RNAiMAX reagent (Invitrogen) according to the manufacturer's instructions, and then further incubated with DC for 24h.

### Cell viability

Huh7 and JNK1- and JNK2-knockout MEF cells seeded in 96-well plates were treated with various concentrations of urushiol derivatives for 48h and then incubated with MTT (0.5 mg/mL) for 2 h. The precipitated formazan product was dissolved in DMSO (Sigma-Aldrich). The absorbance of each well was measured at 560 nm using a microplate reader (BioTek Instruments). The absorbance of control cells was set as 100% viability. Huh7 cells were also treated with DC in presence or absence of Cq (10 μM), 4-PBA (2 mM), and SP600 (20 μM) for 24h prior to the cell viability assay.

### Annexin V/ propidium iodide (PI) staining

Cells treated with DC and/or Cq for 48 h were collected, washed with PBS, and were then prepared according to the Annexin V-FITC Apoptosis Detection Kit instruction protocol (Molecular Probes). The stained cells were analyzed by flow cytometry (BD FACSCalibur^™^ cell analyzer, Becton Dickinson).

### Immunofluorescence

Huh7 cells grown on 8-well chamber slides (Lab-TeK II, Nunc) were fixed with 4% paraformaldehyde, permeabilized with 0.25% Triton X-100, blocked with 10% normal goat serum, and incubated overnight at 4°C with anti-LC3B primary antibody followed by Alexa Fluor^®^ 488, conjugated goat anti-rabbit secondary antibody. Slides were mounted by Vectashield (Vector Laboratories). Images from slides were acquired using a FV500 laser-scanning confocal microscope (Olympus). mCherry-GFP-LC3 stable Huh7 cells were treated with DC and/or Cq for 24h, fixed, and mounted. Images from slides were acquired using a ECLIPSE Ti laser-scanning confocal microscope (Nikon). Quantification for mCherry and GFP puncta was performed in threshold limited images using the ‘Colocalization’ and ‘Analyze Particles’ plugins in ImageJ (NIH).

### RNA analysis

Total RNA was isolated using TRIzol™ (Invitrogen). cDNA was synthesized using ReverTra Ace^®^ qPCR RT kit (Toyobo), quantitative PCR with amplification accomplished using a Mx3000P qPCR System (Agilent Technologies). Primer sequences used in this study were shown in Supplementary Table 1. mRNA levels were normalized for expression of mouse ribosomal protein, Large, P0 (RPLP0) as control and calculated by the comparative threshold cycle method.

### Immunoblotting

Cells were washed with PBS and centrifuged at 1,000 × *g* for 5 min at 4°C. Pellets were resuspended in radioimmunoprecipitation assay (RIPA) buffer with protease and proteasome inhibitors, incubated on ice for 20 min, sonicated, and centrifuged at 20,000 × *g* for 20 min at 4°C. Cell lysates were subjected to SDS-PAGE, followed by transfer to a polyvinylidene difluoride or nitrocellulose membranes (Merck Millipore). Membranes were incubated overnight at 4°C with the specific primary antibodies, followed by incubation with secondary antibodies. Blots were developed using the EZ-Western Lumi Femto™ western blot detection kit (DAEILLAB Service).

### Measurement of necrosis

After DC and/or Cq treatment for 48 h the media were collected and centrifuged at 10,000 × *g* for 5 min at 4°C to remove cells. The supernatant was used for LDH assay (Sigma). For analysis of released HSP90 the supernatant was loaded onto an Amicon Ultra centrifugal filter with a molecular mass cutoff of 3 kDa (Merck Millipore) and centrifuged at 10,000 × *g* for 30 min at 4°C. Concentrated media were removed from the filter, and protein was precipitated with ice-cold acetone. The pellet was subjected to immunoblotting using anti-HSP90.

### Luciferase reporter assay

The human p62 promoter construct (-1,650 to +120) was amplified by PCR using human genomic DNA as template, and cloned into the pGL3-basic firefly luciferase reporter vector (Promega Corp). The construct was verified by DNA sequencing. Huh7 cells seeded in 24-well plate were transfected with pGL3-p62 luciferase reporter using Lipofectamine^®^ 2000 reagent (Invitrogen) for 24h before treatment with DC and/or SP600 for 24h. Cells were harvested and assayed using the Nano-Glo^®^ Dual-Luciferase^®^ Reporter Assay System (Promega Corp). A pNL1.1 luciferase vector (Promega Corp) was used as a normalization control.

### Measurement of intracellular Ca^2+^ mobilization

Cells grown on 4-well chamber slide were treated with 1 μM Fura-2/AM (Invitrogen) in imaging medium for 20 min at 37°C. After washing twice, the cells were incubated with Locke's solution (158.4 mm NaCl, 5.6 mm KCl, 1.2 mm MgCl_2_, 2.2 mm CaCl_2_, 5 mm HEPES, and 10 mm glucose, pH 7.4) and then stimulated with DC. Intracellular Ca^2+^-dependent changes in fluorescence were imaged using an IX81 ZDC (Olympus) inverted microscope equipped with a 40 × oil immersion lens, and the produced at a rate of one image/3s. For ratiometric imaging, cells were alternately excited at 340 nm (Fura-2-Ca^2+^ complex) and 380 nm (free Fura-2) and measured at an emission wavelength of 500 nm. Changes in the F340/F380 nm fluorescence ratio were determined from selected regions across the cytoplasm of a single cell; 10–20 cells were imaged per well, and 30–60 cells were quantified per condition.

### Statistical analysis

All experiments were performed at least three times, and data are presented as mean ± SEM. Differences between the means of the individual groups were assessed by Student's *t*-test or one-way ANOVA; differences were considered significant at *P* < 0.05. The statistical software package, Prism 6.0 (GraphPad Software), was used for these analyses.

## SUPPLEMENTARY MATERIALS FIGURES AND TABLES


