# Supplementary material for: 3-Decylcatechol induces autophagy-mediated cell death through the IRE1α/JNK/p62 in hepatocellular carcinoma cells
Source: Oncotarget. 2017 May 9;8(35):58790–800. doi: 10.18632/oncotarget.17732 (PMC5601693; doi:10.18632/oncotarget.17732)
Supplement: Supplementary file 1 [file oncotarget-08-58790-s001.pdf]

# 3-Decylcatechol induces autophagy-mediated cell death through the IRE1α/JNK/p62 in hepatocellular carcinoma cells

## SUPPLEMENTARY MATERIALS

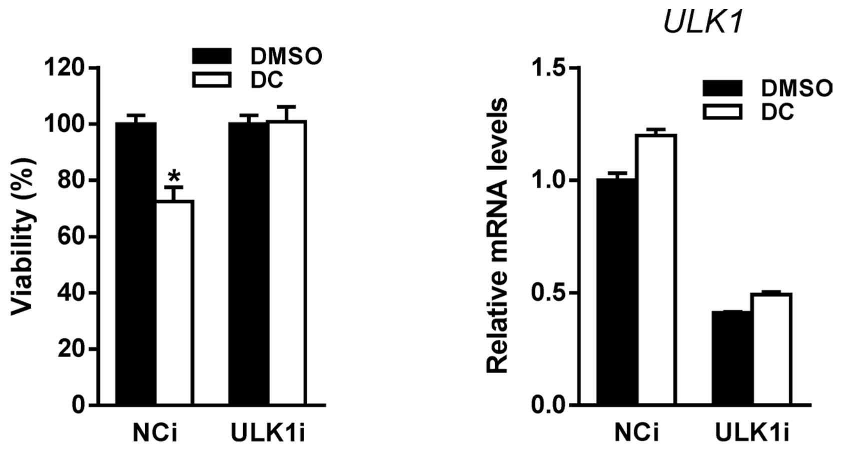


**Supplementary Figure 1: Inhibition of ULK1 prevents DC-induced cell death.** Cells were transfected with siRNA (10 nM) for ULK1 or NCi for 24h, and then 10 µM DC was added and the cell viability and mRNA were measured by MTT assay and RT-qPCR, respectively. **P* <0.05 vs. NCi DMSO. Data are mean ± SEM; n=3.

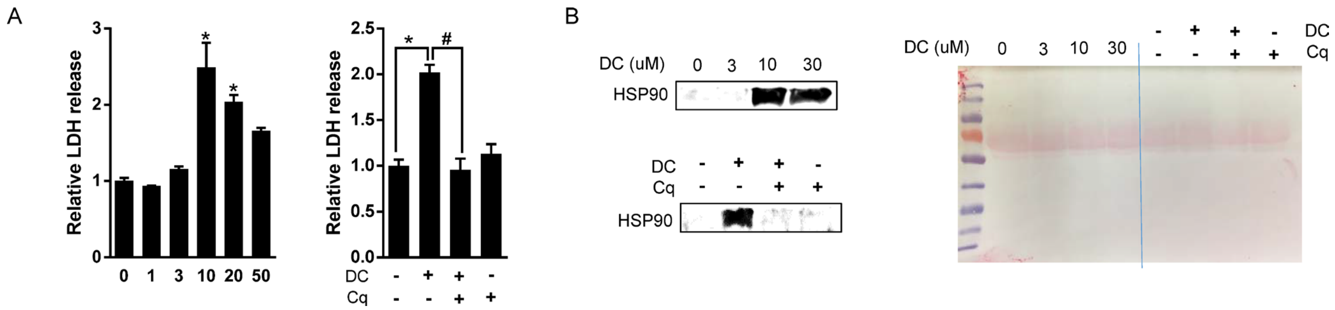


**Supplementary Figure 2: DC induces release of LDH and HSP90.** Cells were treated with DC and/or Cq for 48h, and then the media were collected and analyzed. **(A)** LDH release. *$P$ <0.05 vs. control DMSO; #$P$ <0.05 vs. DC. **(B)** Immunoblot analysis for HSP90.

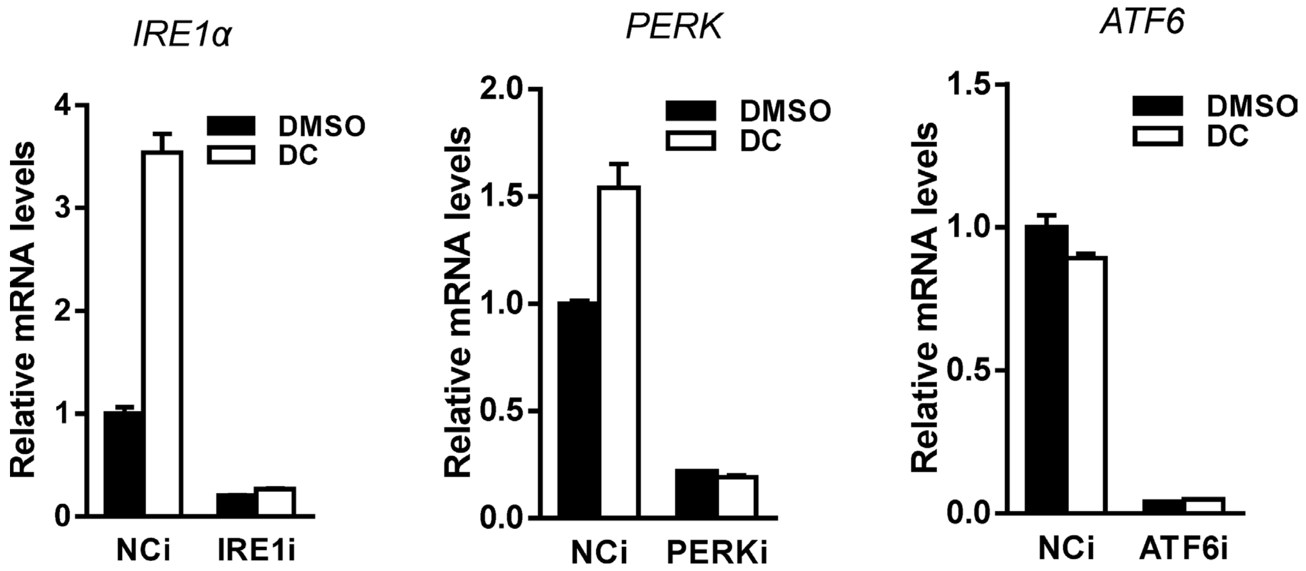


**Supplementary Figure 3: Knockdown efficiency of siRNA targeting each UPR branch.** Cells were transfected with siRNA (10 nM) for PERK, IRE1α, ATF6, or negative control (NCi) for 24h, and then 10 µM DC was added and cells were harvested after 24h. mRNA levels for IRE1α, PERK, and ATF6 were analyzed by RT-qPCR.

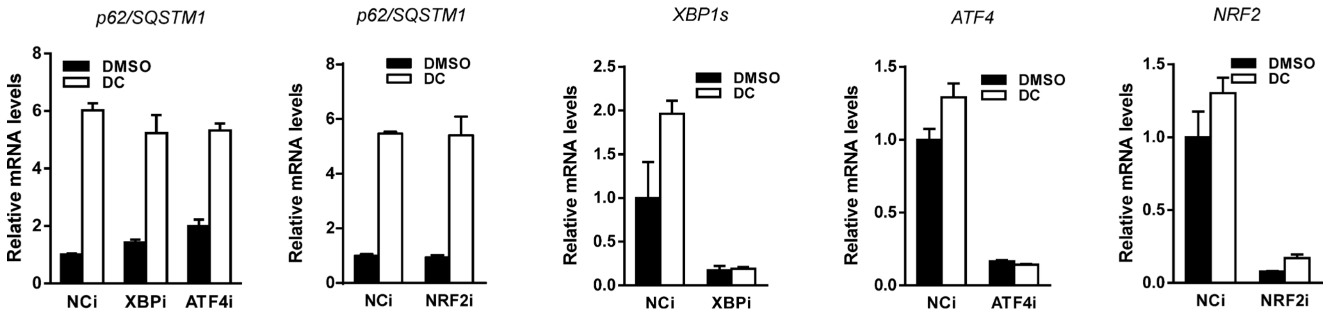


**Supplementary Figure 4: ATF4, XBP1, and NRF2 are not required for DC-induced p62 upregulation.** Cells were transfected with siRNA (10 nM) for ATF4, XBP1, NRF2, or negative control (NCi) for 24h, and then 10 μM DC was added and cells were harvested after 24h. mRNA levels for p62, spliced XBP1 (XBP1s), ATF4, and NRF2 were analyzed by RT-qPCR.

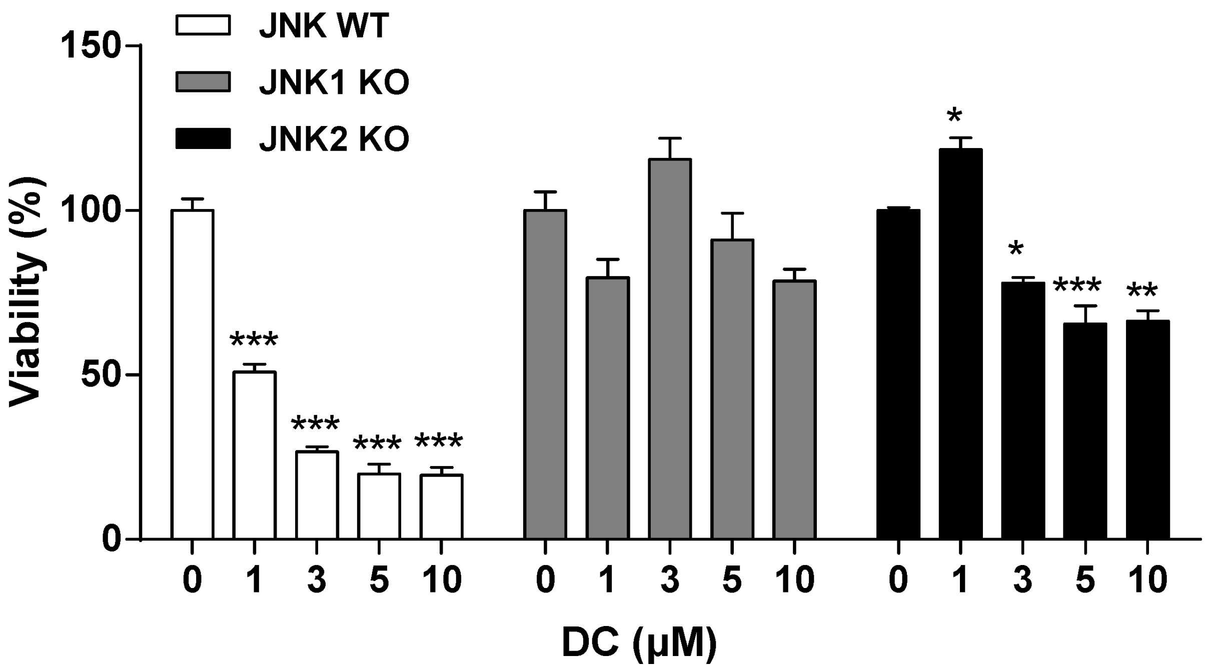


**Supplementary Figure 5: DC induces cell death via JNK pathway.** WT and JNK1- and 2-kcockout MEF cells were treated with DC for 48h. The cell viability was measured by MTT assay. Data are mean ± SEM; n=3. *$P$ <0.05, ** $P$ <0.01, ***$P$ <0.001.

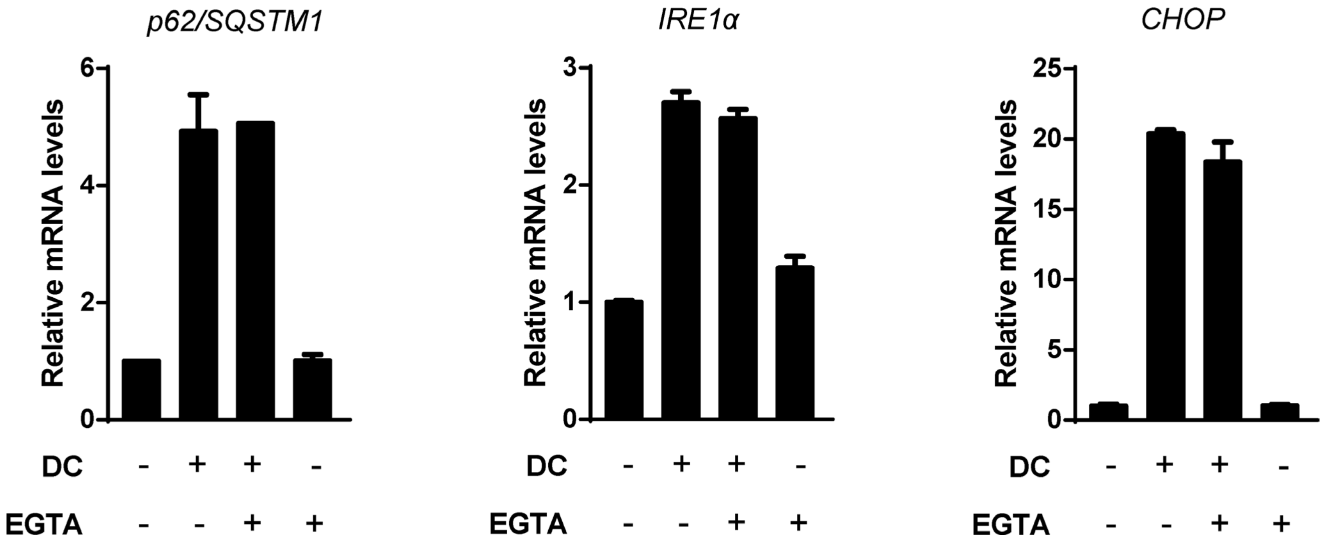


**Supplementary Figure 6: EGTA treatment does not block DC action.** Cells were pretreated with 1mM EGTA for 1h and then exposed to 10 µM DC for 24h. mRNA levels for p62, IRE1α, and CHOP were analyzed by RT-qPCR.

**Supplementary Table 1: Primers used in this study**

| Genes | Forward | Reverse |
|---|---|---|
| **RT-qPCR** | | |
| p62/SQSTM1 | AAGCCGGGTGGGAATGTTG | GCTTGGCCCTTCGGATTCT |
| ATF4 | CCAACAACAGCAAGGAGGAT | GGGGCAAAGAGATCACAAGT |
| ATF6 | TGACAAAGCCCTGATGGTGCTA | TGTTCCAGAGCACCCTGAAGAA |
| CHOP | GGAGAACCAGGAAACGGAAAC | TCTCCTTCATGCGCTGCTTT |
| IRE1α | GCCACCCTGCAAGAGTATGT | ATGTTGAGGGAGTGGAGGTG |
| PERK | CCAGCCTTAGCAAACCAGAG | GTCTTGGTCCCACTGGAAGA |
| XBP1s | GGTCTGCTGAGTCCGCAGCAGG | GGGCTTGGTATATATGTGG |
| NRF2 | AGTGGATCTGCCAACTACTC | CATCTACAAACGGGAATGTCTG |
| ULK1 | AGCACGATTTGGAGGTCGC | GCCACGATGTTTTCATGTTTCA |
| RPLP0 | GTGCTGATGGGCAAGAAC | AGGTCCTCCTTGGTGAAC |
| **RT-PCR** | | |
| XBP1 | TTACGAGAGAAAACTCATGGC | GGGTCCAAGTTGTCCAGAATGC |
| **Cloning** | | |
| p62/SQSTM1 | GGAAGATCTCTGACTCACTGCCGCCAGAC | CTCAAGCTTTGTAGCGAACGCGGAGGC |
